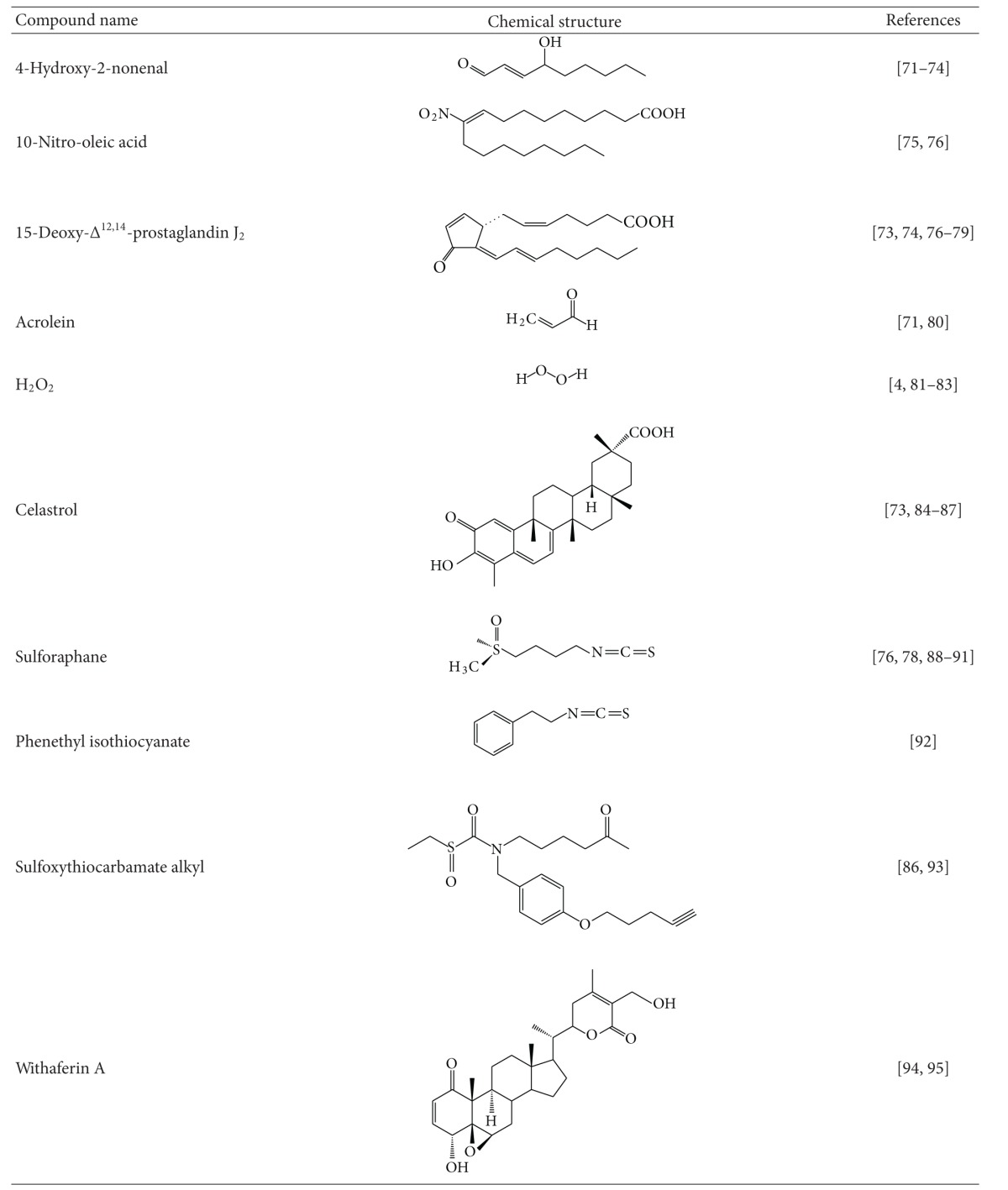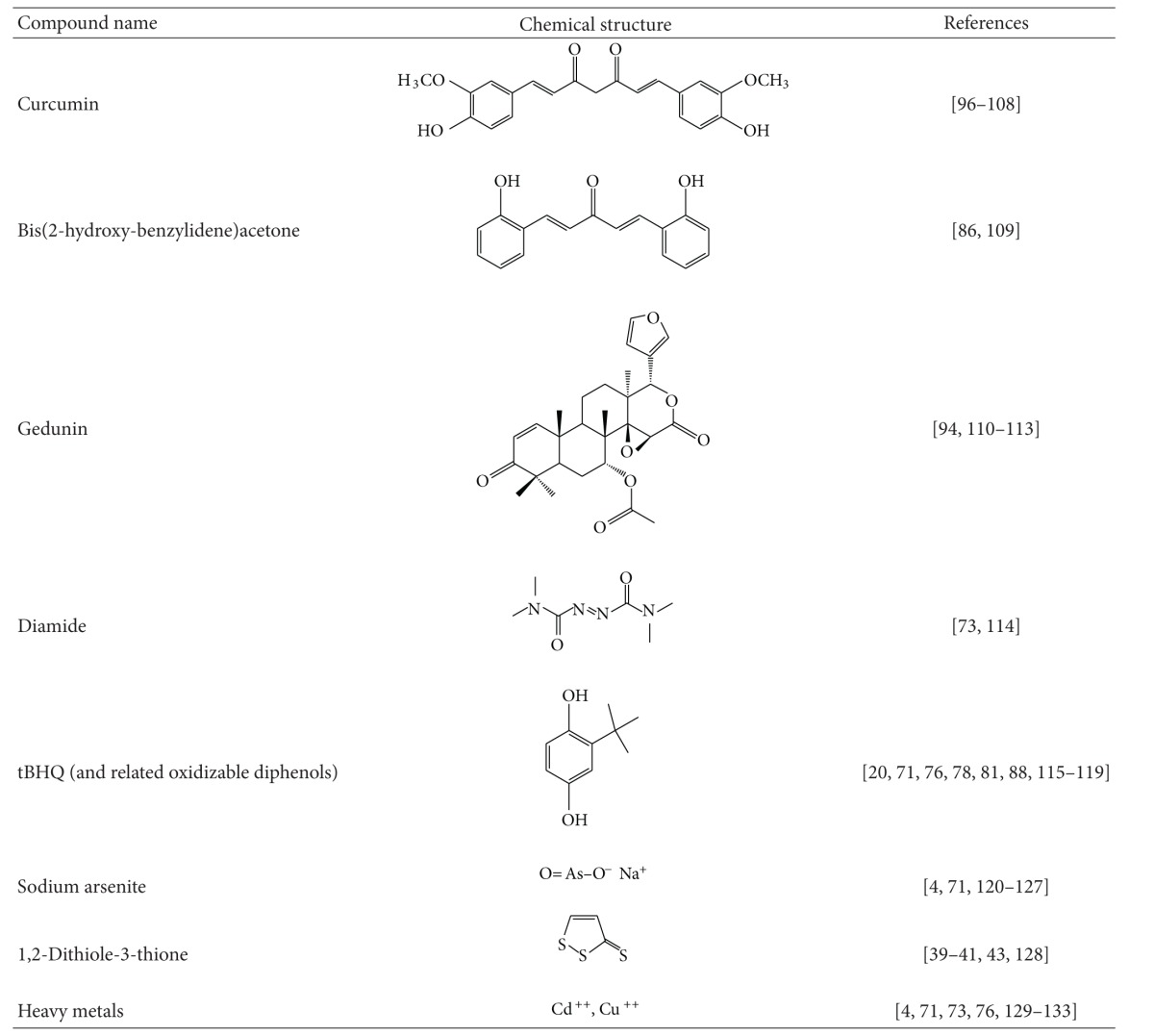# The Role of Sulfhydryl Reactivity of Small Molecules for the Activation of the KEAP1/NRF2 Pathway and the Heat Shock Response

**DOI:** 10.6064/2012/606104

**Published:** 2012-12-23

**Authors:** Albena T. Dinkova-Kostova

**Affiliations:** ^1^Jacqui Wood Cancer Centre, Division of Cancer Research, Medical Research Institute, Ninewells Hospital and Medical School, University of Dundee, James Arrott Drive, Dundee DD1 9SY, UK; ^2^Department of Pharmacology and Molecular Sciences and Department of Medicine, Johns Hopkins University School of Medicine, Baltimore, MD 21205, USA

## Abstract

The KEAP1/NRF2 pathway and the heat shock response are two essential cytoprotective mechanisms that allow adaptation and survival under conditions of oxidative, electrophilic, and thermal stress by regulating the expression of elaborate networks of genes with versatile protective functions. The two pathways are independently regulated by the transcription factor nuclear factor-erythroid 2 p45-related factor 2 (NRF2) and heat shock factor 1 (HSF1), respectively. The activity of these transcriptional master regulators increases during conditions of stress and also upon encounter of small molecules (inducers), both naturally occurring as well as synthetically produced. Inducers have a common chemical property: the ability to react with sulfhydryl groups. The protein targets of such sulfhydryl-reactive compounds are equipped with highly reactive cysteine residues, which serve as sensors for inducers. The initial cysteine-sensed signal is further relayed to affect the expression of large networks of genes, which in turn can ultimately influence complex cell fate decisions such as life and death. The paper summarizes the multiple lines of experimental evidence demonstrating that the reactivity with sulfhydryl groups is a major determinant of the mechanism of action of small molecule dual activators of the KEAP1/NRF2 pathway and the heat shock response.

## 1. Introduction

One of the least abundant amino acids in all organisms, cysteine, has unique chemical features which nature uses in multifaceted ways. The chemical reactivity and plasticity of the sulfhydryl group of cysteine are in the heart of many molecular phenomena such as enzyme-based catalysis, metal coordination, protein folding, and protein-protein interactions. This versatility in turn affects some of the most fundamental biological processes such as cell division, proliferation, differentiation, apoptosis, cell signaling, and responses to oxidative, nitrosative, and electrophilic stress. In addition, *S*-thiolation, *S*-nitrosation, and the formation of disulfide bonds and sulfenic acid, or the so-called “reactive sulfur species” [1], that occur within cysteine-containing proteins can further affect the function of many other proteins that associate with the host molecule which harbours such modifications. Furthermore, the complex relationships between cysteine-containing redox-regulated proteins and their interacting networks, collectively termed as “the cellular thiolstat,” have essential contributions to cell fate decisions [2]. It is therefore perhaps not surprising that sulfhydryl-reactive small molecules, both naturally occurring and synthetic, can profoundly alter a wide range of physiological processes by reacting with cysteine residues within their protein targets. This paper focuses on the importance of sulfhydryl reactivity of such small molecules for activation of two quintessential cytoprotective mechanisms: the KEAP1/NRF2 pathway and the heat shock response.

## 2. The KEAP1/NRF2 Pathway

**Figure 1:**
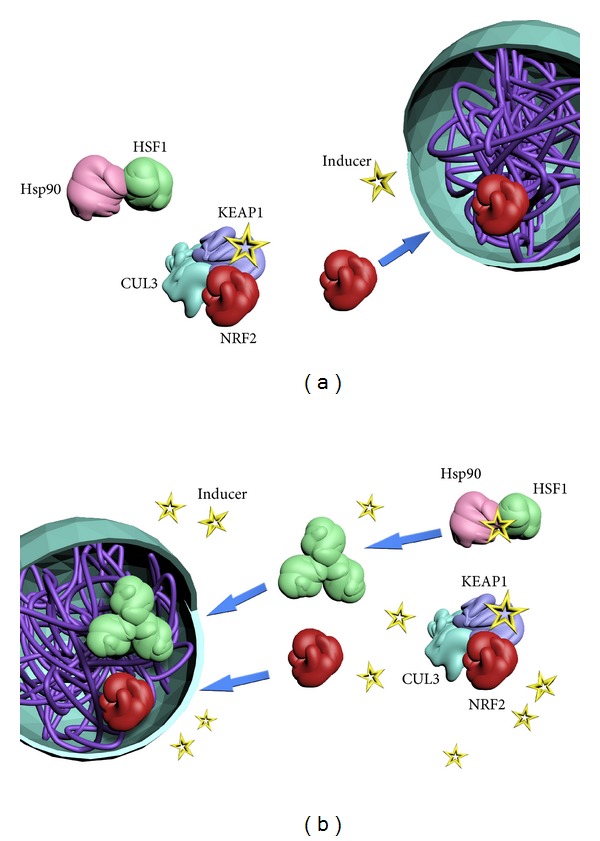
The KEAP1/NRF2 pathway and the heat shock response are highly inducible essential defense systems that allow the cell to adapt and survive under various conditions of stress by regulating the expression of elaborate networks of several hundred genes with versatile cytoprotective functions. At low concentrations (a), inducers (yellow stars) bind to cysteine residues of the protein sensor KEAP1 (purple). Consequently, KEAP1 loses its ability to target transcription factor NRF2 (red) for ubiquitination via CUL3 (cyan)-dependent E3 ubiquitin ligase, preventing subsequent proteosomal degradation of NRF2 and allowing its accumulation, nuclear translocation, and binding to the ARE as a heterodimer with a small MAF protein (not shown) to induce expression of NRF2-target genes. At high concentrations (b), in addition to activating the KEAP1/NRF2 pathway, inducers also activate the heat shock response by chemically reacting with transcription factor HSF1 (green), or a negative regulator of HSF1, such as Hsp90 (pink) or Hsp70 (not shown). As a result, HSF1 dissociates from the protein complex, trimerizes, undergoes complex posttranslational modifications, translocates to the nucleus, and induces expression of HSF1-target genes.

The KEAP1/NRF2 pathway, also known as “the phase 2 response” [3] or “the electrophile counterattack response” [4], is an essential cellular defense mechanism which regulates the expression of more than 500 genes [5]. Transcription factor nuclear factor-erythroid 2 p45-related factor 2 (NRF2), which operates as an obligatory heterodimer with a small MAF transcription factor, is the main orchestrator of this transcriptional program [6]. The predominant way of regulation of the levels of NRF2 within the cell is by control of its protein stability. At basal state, NRF2 is a very unstable protein with a half-life of just a few minutes [7] as it is continuously targeted for ubiquitination and proteasomal degradation by the repressor protein Kelch-like ECH-associated protein 1 (KEAP1) [8], which functions as a substrate adaptor for Cullin-3- (Cul3-) based E3 ubiquitin ligase (Figure 1(a)) [9–11]. Recently, another pathway of KEAP1-independent regulation of NRF2 was uncovered, by the concerted action of glycogen synthase kinase-3*β* (GSK3*β*) and *β*-transducin repeat-containing protein (*β*-TrCP) which serves as a substrate adaptor for Cullin-1-(Cul1-) based E3 ubiquitin ligase [12–14]. Several different models have been proposed to explain the intricate mechanistic details of operation of the KEAP1/NRF2 pathway under basal as well as induced conditions, and these have been recently reviewed [15–17]. One common feature of these models is that, in addition to being a repressor and a substrate adaptor protein for the ubiquitination of NRF2, KEAP1 is also the sensor for inducers. This inducer-sensing capability of KEAP1 is due to its highly reactive cysteine residues which chemically interact with inducers of many different types and structures [18–21], leading to the loss of its substrate adaptor function. The loss of function of KEAP1 leads to stabilization of NRF2, nuclear accumulation of the transcription factor, and activation of the expression of genes that have antioxidant response elements (AREs), also known as electrophile-responsive elements (EpRE), in their promoter regions with the core consensus sequence 5′-TGABnnnGC-3′ (where B = C, or G, or T, and the letter “n” represents any nucleotide) [22–29].

The ARE-containing NRF2-dependent genes encode a large network of proteins with versatile cytoprotective functions. The earliest recognized ARE-dependent genes were those which participate in the metabolism and transport of endo- and xenobiotics, such as glutathione *S*-transferases (GSTs), NAD(P)H:quinone oxidoreductase 1 (NQO1), epoxide hydrolase, and UDP glucuronosyltransferases [26–34]. Induction of these enzymes is also accompanied by elevation in glutathione levels [31], due to the ARE- and NRF2-dependent transcriptional upregulation of both the heavy and the light subunits of the heterodimeric *γ*-glutamylcysteine ligase, the enzyme which catalyzes the rate-limiting step in the *de novo* biosynthesis of glutathione [35–37]. The identification of multiple functional AREs in the upstream regulatory regions of heme oxygenase 1 (HO-1) added this enzyme to the group of ARE-dependent gene products [38]. The use of differential hybridization methodology revealed that the upregulation of the already known ARE-dependent genes was also accompanied by the coordinate expression of genes encoding the heavy and light subunits of ferritin as well as anti-inflammatory enzymes such as leukotriene B_4_ dehydrogenase [39–41]. Global gene expression profiling confirmed previous findings and added new knowledge regarding the role of NRF2 in maintaining the cellular redox homeostasis and providing reducing equivalents. It became clear that several enzymes that participate in the synthesis, utilization, and regeneration of glutathione, thioredoxin, and NAD(P)H are also regulated by this transcription factor, including *χ*-CT, the core subunit of the cystine/glutamate membrane transporter, *γ*-glutamylcysteine ligase catalytic and modulatory subunits, glutathione reductase, thioredoxin, thioredoxin reductase, glucose 6-phosphate dehydrogenase, 6-phosphogluconate dehydrogenase, and malic enzyme [42–44]. Interestingly, the expression of genes which code for proteins that participate in the repair and removal of damaged proteins, such as subunits of the 26S proteasome, was also found to be NRF2 dependent [43]. Most recently, it was discovered that NRF2 contributes to the regulation of purine biosynthesis by controlling the expression of genes encoding enzymes in the pentose phosphate pathway [45, 46]. By integrating chromatin immunoprecipitation with parallel sequencing (ChIP-Seq) and global transcription profiling, Malhotra et al. identified 645 basal and 654 inducible direct targets of NRF2, with 244 genes at the intersection [5]. Thus, the number and the functional diversity of the NRF2-dependent cytoprotective proteins is extraordinary and provides the cell with multiple defense mechanisms.

The generation of NRF2-knockout mice [6, 47] confirmed that NRF2 is the major orchestrator of the cellular stress response to oxidants and electrophiles. Indeed, NRF2-knockout cells and animals are much more sensitive to the damaging effects of electrophiles, oxidants, and inflammatory agents in comparison to their wildtype counterparts; conversely, pharmacological or genetic activation of NRF2 has protective effects in numerous models of chronic disease, including cancer [29, 48, 49]. The failure of the NRF2-knockout mice to adapt to conditions of stress results in greatly accelerated disease pathogenesis in comparison to wildtype animals [50, 51]. However, the cytoprotective properties of the KEAP1/NRF2 pathway can be exploited by tumor cells to promote their survival. Indeed, NRF2 is frequently constitutively activated in established tumors. Mutations in *KEAP1* or *NRF2*, which abrogate formation of the complex and lead to NRF2 accumulation and constitutive upregulation of the pathway, have been detected in several types of human cancer, including cancer of the lung, oesophagus, ovary, gallbladder, and skin [52–57]. A very recent comprehensive genomic characterisation study on squamous cell lung cancer conducted by the Cancer Genome Atlas Research Network reported the occurrence of mutations in *NRF2*, *KEAP1*, or *CUL3 *in 34% of 178 lung squamous cell carcinomas [58]. In addition, under conditions of oncogenic stress, such as that occurring during permanent activation of oncogenic K-RAS and B-RAF, activation of NRF2 can facilitate cell proliferation [59]. It was also recently shown that when PI3 K-AKT signalling is sustained, NRF2 redirects glucose and glutamine into anabolic pathways, and thus affects primary metabolism [45]. Such conditions have the potential to favor tumor development and, coupled with the increased activity of detoxification enzymes, to also decrease therapeutic efficacy and contribute to drug resistance. This double-edged nature of the consequences of either downregulating or upregulating the KEAP1/NRF2 pathway poses a major challenge for the development and implementation of inducers or inhibitors of this pathway as chemopreventive and/or chemotherapeutic agents.

## 3. The Heat Shock Response

Another essential inducible defense mechanism is the heat shock response which protects the cell under conditions of acute and chronic proteotoxic stress and preserves the integrity of the proteome. The central regulator of the heat shock response is transcription factor heat shock factor 1 (HSF1) [60, 61]. Under basal conditions, HSF1 is an inactive monomeric phosphoprotein bound to heat shock protein 90 (Hsp90) (Figure 1(b)). Upon activation of the heat shock response by thermal stress or small-molecule inducers, HSF1 dissociates from the Hsp90 complex and forms a DNA binding-competent trimer which binds to heat shock elements (HSEs, consensus inverted repeat sequences nGAAn) in the regulatory regions of its target genes and activates their transcription [62, 63]. HSF1 undergoes a number of posttranslational modifications, such as phosphorylation, sumoylation, and acetylation, all of which have been implicated in regulating its transcriptional activity. This transcription factor is also negatively feedback regulated by the products of the genes whose expression it regulates, that is, heat shock proteins, such as Hsp70 and Hsp40. 

By the use of differential display, transcriptional profiling, and proteomic approaches, it has been shown that, depending on the experimental system, the number of proteins whose gene expression is induced by heat shock ranges between 50 and 200 [64] and includes molecular chaperones that prevent unspecific aggregation of nonnative or partially misfolded proteins (e.g., Hsp70 and Hsp40), proteolytic proteins that participate in the removal of irreversibly damaged proteins (e.g., BAG3 (BCL2-associated protein), APG5L (protein involved in autophagy), CASP1 (caspase, cysteine protease), NEDD4L (neural precursor cell-expressed developmentally downregulated 4 like, ubiquitin-protein ligase)), RNA- and DNA-modifying enzymes, which participate in the repair of damaged DNA (e.g., the bacterial DNA glycosylase MutM), metabolic enzymes that maintain the energy supply of the cell [e.g., ACAT2 (acetyl-CoA acetyltransferase), ALAS1 (aminolevulinate synthase), ChGn (chondroitin *β*-1,4-*N*-acetylgalactosaminyltransferase], transcription factors, kinases, or phosphatases that further activate other stress response pathways [e.g., RHOH (Ras homolog), PTPG1 (tyrosine phosphatase), RGS2 (regulator of G-protein signaling), IER5 (regulator of immediate early response)], proteins involved in sustaining cellular structures such as the cytoskeleton and membranes (e.g., TJP4 (tight junction associated protein), SIPA1L3 (signal-induced proliferation-associated 1-like protein 3)), and proteins involved in transport and detoxification (e.g., the amino acid transporter SLC38A2).

Similar to the case of the KEAP1/NRF2 pathway, activation of HSF1 commonly occurs in cancer. Recent work has shown that, in malignant tumors, HSF1 is responsible for the orchestration of a transcriptional program, termed the HSF1 cancer program, which is distinct from the transcriptional response that is induced by heat shock. This program includes both HSF1 positively and negatively regulated genes and drives the expression of cancer-specific genes supporting oncogenic processes such as cell-cycle regulation, mitosis, energy metabolism, translation, cell signalling, and adhesion [65]. Moreover, this HSF1 cancer program was found to be very significantly and broadly associated with poor outcomes such as metastasis and death in breast cancer, whereby patient survival decreases dose dependently as HSF1 nuclear levels increase [65, 66]. Further analysis of multiple independent gene expression data sets from patients with known clinical outcomes has revealed that activation of the HSF1 cancer program is also associated with reduced survival in colon and lung cancer, and, importantly, much more significantly than any individual, or even a panel of, *HSP* transcript(s) [65]. These findings demonstrate the very broad role of HSF1 which extends beyond the control of gene expression of heat shock proteins and the heat shock response and impacts cell survival in multiple ways.

## 4. Reactivity with Sulfhydryl Groups: A Common Chemical Property of Small Molecule Dual Activators of the KEAP1/NRF2 Pathway and the Heat Shock Response

The KEAP1/NRF2 pathway and the heat shock response are distinct cytoprotective mechanisms that are regulated by independent transcription factors which orchestrate specific transcriptional programs. Curiously, however, many small molecules which have been found to activate one of these pathways were independently shown to induce the other (Table 1). Examples include the endogenous 4-hydroxy-2-nonenal, nitro-fatty acids, 15-deoxy-Δ^12,14^-prostaglandin J_2_, acrolein, and hydrogen peroxide; the naturally occurring phytochemicals celastrol, sulforaphane, withaferin A, gedunin, and curumin; as well as the synthetic bis(benzylidene)alkanones, sulfoxythiocarbamates, oxidizable diphenols, and diamide. Although such compounds are able to participate in chemical reactions that are not limited to those with sulfhydryl groups, sulfhydryl reactivity (by oxidation-reduction, alkylation, or thiol-disulfide interchange) is their only common attribute. Seminal studies by Paul Talalay and his colleagues led to the recognition of this chemical property as a unifying feature of inducers of the phase 2 response [67]. It was then hypothesized that the protein sensor for inducers, which many years later was identified as KEAP1, must contain highly reactive cysteine residues. The development and use of a microtiter-based bioassay in the murine hepatoma Hepa1c1c7 cell line for screening of potential inducers of the NRF2-target gene NQO1 [68, 69] further strengthened the concept that sulfhydryl reactivity constitutes the only common property of both naturally occurring as well as synthetic inducers. NQO1 inducers were grouped in 10 distinct chemical classes: (i) Michael acceptors (olefins or acetylenes conjugated with electron-withdrawing groups), (ii) oxidizable diphenols and diamines, (iii) conjugated polyenes, (iv) hydroperoxides, (v) trivalent arsenicals, (vi) heavy metals, (vii) isothiocyanates, (viii) dithiocarbamates, (ix) dithiolethiones, and (x) vicinal dimercaptans [70].

In a large series of semisynthetic pentacyclic triterpenoids, the presence of the 1-en-3-one functional group (i.e., Michael acceptor) in the A-ring was found to be essential for inducer activity, and the introduction of a cyanoenone functionality, which strengthens the electrophilicity, further increased the inducer potency [134, 135]. These experimental findings were also supported by molecular orbital calculations [136]. More recently, tricyclic and monocyclic cyanoenone derivatives were synthesized; some of which are extremely potent and remarkably bioavailable inducers in cell systems as well as *in vivo* in animals [19, 21, 137, 138]. Oxidizable diphenols (such as tBHQ and catechol estrogen metabolites) represent another class of inducers, and activation of NRF2 by these compounds requires oxidation to their corresponding quinone derivatives [20, 139, 140], which then react with cysteine residues of KEAP1 [20, 71, 81, 88, 141]. In MCF-7 human breast cancer cells stably transfected with an ARE-luciferase reporter (AREc32 cells), tBQ and estradiol-3,4-quinone induce ARE-dependent gene expression [20]. In contrast, short-term (30 min) exposure to tBHQ, 2-hydroxyestradiol, 4-hydroxyestradiol, or 4-hydroxyestrone has a much more modest effect on the luciferase gene expression. Importantly, however, induction by these oxidizable diphenols is greatly potentiated (by ~4-5-fold) under conditions that favor formation of the corresponding quinones, that is, in the presence of transition metals and O_2_, strongly suggesting that the electrophilic quinone metabolites are the ultimate inducers. Trivalent arsenicals, such as phenylarsine oxide and sodium arsenite, react readily with vicinal sulfhydryl groups to form highly stable five-membered cyclic products [142] and are potent NRF2 activators [4]. Two recent high-throughput screens, one employing an ARE-dependent luciferase reporter to screen 1.2 million compounds in human neuroblastoma IMR-32 cells [143] and another utilizing a luciferase reporting on the stabilization of NRF2 which evaluated 20,000 biologically active compounds in human neuroblastoma SH-SY5Y-Neh2-luc cells [110], have confirmed that sulfhydryl reactivity is a prominent feature of inducers of the KEAP1/NRF2 pathway. One of the most potent inducers identified in the ARE-reporter assay, a chloroquinolone derivative, has an electrophilic aryl chloride group located at the *β*-position of two *α*,*β*-unsaturated carbonyl moieties, which could potentially be displaced by a nucleophilic sulfhydryl group through a 1,4-addition-elimination reaction. Gedunin, a natural product identified as a new inducer in the NRF2-stabilization luciferase reporter assay has two electrophilic centers and, interestingly, had previously been identified as an activator of HSF1 and an inducer of Hsp70 [111]. 

Reactivity with sulfhydryl groups is also emerging as a common property of small molecule inducers of the heat shock response. Activation of HSF1 and enhanced transcription of Hsp70 have been observed upon exposure of cells to heavy metals such as Cd^++^ and Cu^++^ [129–133], as well as alkylating agents such as iodoacetamide [144], the anticancer drugs 1,3-bis-(2-chloroethyl)-1-nitrosourea, 1-(2-chloroethyl)-3-cyclohexyl-1-nitrosourea [145, 146], and nephrotoxic cysteine conjugates-derived reactive electrophilic metabolites [147]. The trivalent arsenical sodium arsenite activates HSF1 and induces heat shock proteins [120–127]. The isothiocyanates sulforaphane and phenethyl isothiocyanate induce heat shock proteins in cultured cells [89, 148] and *in vivo *in animals that had been administered these agents [90, 92]. The double Michael acceptor-containing curcumin, the principal coloring and flavoring agent of curry, is also an inducer of heat shock proteins [96–102]. Hepatic murine Hsp70 and Hsp40 were induced 24 h after treatment with 1,2-dithiole-3-thione, and this induction was observed in livers of both wildtype as well as NRF2-knockout mice [128]. Curiously, pyrrolidine dithiocarbamate, which can react with sulfhydryl groups by thiol-interchange reactions, also activates HSF1 [149, 150]. The electrophilic oxidized and nitrated lipids such as 4-hydroxy-2-nonenal, 15-deoxy-Δ^12,14^-prostaglandin J_2_, and 10-nitro-octadecenoic acid have all been shown to induce the HSF1-mediated heat shock response. Thus, exposure of human colon cancer cells to 4-hydroxy-2-nonenal caused nuclear accumulation of HSF1 and induced endogenous Hsp70 and Hsp40, as well as a luciferase reporter under the transcriptional control of the consensus HSE, and silencing of the expression of HSF1 by siRNA abolished this induction [72]. In a myocardial ischemia and reperfusion model in male Wistar rats, 15-deoxy-Δ^12,14^-prostaglandin J_2_ enhanced DNA-binding activity of HSF1 and induced the expression of Hsp70 [77]. Genome-wide transcriptional profiling of human endothelial cells after treatment with 10-nitro-octadecenoic acid revealed that the heat shock response is the major pathway activated by this nitro-fatty acid [75]. Similarly, induction of heat shock proteins, such as Hsp70 and Hsp40, was found to be a characteristic feature of the transcriptional response of A459 human lung cancer cells to the electrophilic *α*,*β*-unsaturated aldehyde acrolein [80]. The quinone methide-containing natural product celastrol is a potent inducer of the heat shock response [84, 85], and this electrophilic moiety has been preserved during the design and synthesis of celastrol analogues as affinity probes for identification of its protein targets [151]. Other phytochemicals which have been shown to induce the heat shock response, such as sulforaphane, withaferin A, gedunin, and curcumin, also have the ability to react with sulfhydryl groups. Curiously, high concentrations of 17*β*-estradiol activate HSF1 and induce Hsp70 [115, 116], and it could be speculated that, similar to the activation of NRF2, the actual activators of HSF1 could be the electrophilic quinone derivatives of the catechol metabolites of 17*β*-estradiol, 2-hydroxy and 4-hydroxyestradiol [152]. A high-throughput screen for HSF1 activators in which a library of more than 80,000 compounds was tested identified the presence of an *α*,*β*-unsaturated carbonyl moiety as an essential structural requirement for induction of the heat shock response [94]. Among the active compounds were various natural products such as the limonoids, anthothecol, cedrelone, gedunin, and 7-desacetoxy-6,7-dehydrogedunin, the macrocyclic lactone dehydrocurvularin, and the steroidal lactone withaferin A, as well as celastrol, all of which have an *α*,*β*-unsaturated carbonyl functional group. A similar finding was made in an even larger scale study of approximately 1 million compounds, in which electrophilicity was found to be a common characteristic of the ~200 small molecule activators of the heat shock response that were identified [132]. 

To test the functional importance of sulfhydryl reactivity for activation of the KEAP1/NRF2 pathway and the heat shock response, inducers of these pathways have been used in combination with classical nucleophiles. One example is the quinone methide celastrol. Nucleophiles, such as cysteine and glutathione, participate in reversible regioselective and stereospecific addition reactions with celastrol to form Michael adducts, such that the nucleophilic attack is favored exclusively at the *β*-face with the nucleophile approach *syn* to the *β*-C9 methyl [153]. The ability of a biotinylated derivative of celastrol to bind to tubulin in cells is abolished in the presence of dithiothreitol (DTT), demonstrating the importance of the electrophilic moiety of celastrol for reaction with its intracellular protein targets [151]. In agreement, induction of both NRF2- and HSF1-dependant genes by celastrol are prevented by incubation with DTT or *N*-acetylcysteine [84, 132], and the ability of the quinone methide to induce Hsp70, downregulate Cdk4 and Cyclin D1, and cause cell cycle arrest is reversed by *N*-acetylcysteine and reduced glutathione, but not vitamin C or oxidized glutathione [154]. Similarly, activation of HSF1 by both diamide and 15d-PGJ_2_ is completely abolished by incubation of these compounds with a 5-fold or greater excess of DTT prior to introducing them into the cell culture medium [73]. DTT also blocks the induction of an hsp70-luciferase reporter by sodium arsenite [120]. Whereas treatment with pyrrolidine dithiocarbamate caused dose- and time-dependent HSF activation, this activity is significantly inhibited by *N*-acetylcysteine and completely abolished by DTT [149]. The simultaneous addition of *N*-acetylcysteine and withaferin A to a reporter system in which the expression of enhanced green fluorescent protein is controlled by a minimal consensus HSE-containing promoter suppresses heat shock activation almost completely [94]. In the case of the NRF2 activation, elevating the intracellular glutathione levels by the inducer sulforaphane lowers, whereas depletion of glutathione by inhibiting its biosynthesis with buthionine sulfoximine increases the inducer potency of oxidizable diphenols [20]. 

Evaluation of structural analogues of small molecule activators of NRF2 and/or HSF1 which either lack the electrophilic center(s) or have decreased electrophilicity has also supported the notion of the importance of sulfhydryl reactivity for induction of the KEAP1/NRF2 pathway and the heat shock response. In all cases, such alterations have led to either a complete loss of activity or a decrease in inducer potency. Thus, the sulfoxythiocarbamate group-containing analogues of sulforaphane which have much weaker sulfhydryl reactivity in comparison with the isothiocyanate group of sulforaphane are also less potent (5–10-fold) inducers of the NRF2-dependent enzyme NQO1 [93]. Among several series of naturally occurring and synthetic phenylpropanoids, the presence of a hydroxyl group at the *ortho*-position on the phenyl ring increases the chemical reactivity with sulfhydryl groups in parallel with enhancement of the inducer potency [103, 109, 155]. Compared to its hydroxylated analogue, bis(2-hydroxybenzylidene)acetone, the closely structurally related bis(benzylidene)acetone, reacts much more slowly with the sulfhydryl groups of glutathione and DTT and has much lower potency in inducing both the KEAP1/NRF2 pathway and the heat shock response [86, 109]. The recognition that inducer potency parallels sulfhydryl reactivity was a major driving force for the formation of the concept that the interaction with inducers and their intracellular sensor protein is based on chemical reactivity with cysteine residue(s) and not on receptor-ligand type of binding [4, 67, 109, 156].

Oleanolic acid which has the same pentacyclic skeleton, but lacks the electrophilic centers of the semisynthetic enone- or cyanoenone-bearing triterpenoids which induce NQO1 at nanomolar concentration, is completely inactive in this assay [134]. The cyclopentenone prostaglandins, which contain an *α*,*β*-unsaturated carbonyl moiety in the cyclopentane ring, activate both HSF1 and NRF2, whereas other arachidonic acid metabolites that lack this electrophilic functional group do not have these effects [74, 157, 158]. In contrast to the large number of HSF1- and NRF2-target genes that are induced upon exposure to nitro-oleic acid, no induction is observed when cells are treated with its nonelectrophilic counterpart, oleic acid [75]. An analog of withaferin A, 2,3-dihydrowithaferin A, which lacks the *α*,*β*-unsaturated carbonyl moiety, is inactive in inducing HSF1 [94]. Similarly, the withanolide analogs, pubesenolide and viscosalactone B which lack this sulfhydryl-reactive functional group, and curvularin which only differs from the active compound dehydrocurvularin by the absence of the *α*,*β*-unsaturated carbonyl moiety, are also inactive [94]. Taken together, these findings imply that although not all small molecules that have the ability to react with sulfhydryl groups are inducers, reactivity with sulfhydryl groups constitutes the critical chemical signature responsible for induction of both cytoprotective pathways. 

## 5. Implications of Sulfhydryl Reactivity of Inducers of the KEAP1/NRF2 Pathway and the Heat Shock Response for the Identity of Their Protein Targets

**Figure 2:**
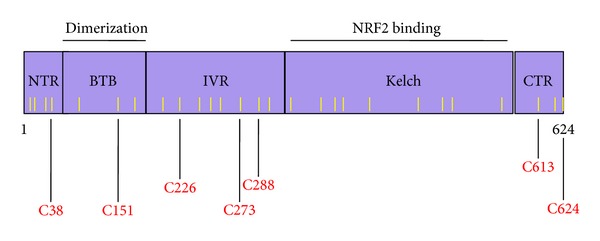
Domain structure of human KEAP1. NTR: N-terminal region (amino acids 1−60); BTB: broad complex, Tramtrack, Bric-á-brac (amino acids 61–179). KEAP1 forms a homodimer through the BTB binding domain; IVR: intervening region (amino acids 180–314); Kelch domain (amino acids 315–598). The Kelch domain is the binding site with NRF2; CTR: C-terminal region (amino acids 599–624). The positions of the cysteine residues are indicated with yellow bars. The most commonly modified cysteine residues by sulfhydryl-reactive small molecules are shown in red.

The common chemicals signature of reactivity with sulfhydryl groups among inducers of the KEAP1/NRF2 pathway and the heat shock response indicates that the protein targets for inducers of these pathways must possess reactive cysteine residues. The negative regulator of NRF2, KEAP1, is a multidomain homodimeric protein which has five distinct domains (Figure 2): (i) NTR: N-terminal region (amino acids 1−60); (ii) BTB: broad complex, Tramtrack, Bric-á-brac (amino acids 61−179)—the domain through which KEAP1 dimerizes; (iii) IVR: intervening region (amino acids 180−314) which is a particularly cysteine-rich region containing 8 cysteine residues among its 134 amino acids; (iv) Kelch domain (amino acids 315−598)—the domain through which KEAP1 binds to NRF2 and CUL3; and (v) CTR: C-terminal region (amino acids 599−624). There are 25 and 27 cysteine residues among the 624 amino acids of murine and human KEAP1, respectively. Nine of these cysteine residues (i.e., C23, C38, C151, C241, C273, C288, C297, C319, and C613) are flanked by basic amino acids and therefore predicted to have *pK*_*a*_ values lower than that of free cysteine, favouring the formation of the thiolate anion at neutral pH, and thus increasing the cysteine reactivity [159]. The NRF2 inducers sulforaphane and its sulfoxythiocarbamate derivative STCA have been shown to react with cysteine residues of KEAP1 using both purified recombinant protein as well as ectopically expressed KEAP1 isolated from cells that had been exposed to these compounds [18, 71, 93, 160, 161]. Mass spectroscopy and mutagenesis analyses have established that C151 in the BTB domain, and C273 and C288 in the IVR domain are critical for the repressor function of KEAP1; however, depending on the reaction conditions and the experimental system used, other cysteine residues have been also reported to be modified by sulforaphane, such as C38 in the N-terminal domain, C368, C489, and C583 in the Kelch domain, and C624 in the CTR [160, 161]. KEAP1 reacts with *tert*-butyl quinone (tBQ), and the UV-Vis spectrum of the reaction product is identical to that of the product of the reaction of tBQ and DTT, implying that tBQ modifies cysteine residues of KEAP1 [20]. Mutation of C151 results in KEAP1 being a constitutive repressor of NRF2, which is unresponsive to activation by sulforaphane or tBHQ [9, 88]. Substitution of C273 or C288 with either serine or alanine leads to a complete loss of repressor activity, and KEAP1 is unable to repress NRF2 even under basal conditions [74, 88, 162]. Transgenic mice expressing either C273A or C288A KEAP1 mutants have confirmed that these residues are required for the repressor function of KEAP1 under basal conditions [163].

The cysteine residues of KEAP1 form multiple discrete sensors for inducers. In a study conducted in zebrafish, a series of activators of NRF2 were classified into two groups: (i) inducers which react with C151 of KEAP1 (e.g., sulforaphane) and (ii) inducers which react with C273 (e.g., 15-deoxy-Δ^12,14^-prostaglandin J_2_) [78]. Transgenic complementation rescue assays using embryonic fibroblasts and primary peritoneal macrophages isolated from mice expressing the C151S mutant of KEAP1 which were exposed to various inducers further confirmed these findings [76]. It was shown that tBHQ, diethylmaleate, sulforaphane, and dimethylfumarate are sensed primarily by C151 of KEAP1, whereas the sensor(s) for 15-deoxy-Δ^12,14^-prostaglandin J_2_, 2-cyano-3,12 dioxooleana-1,9 diene-28-imidazolide (CDDO-Im), ebselen, nitro-oleic acid, and cadmium chloride are C151 independent. Recombinant KEAP1 reacts with monocyclic, tricyclic, and pentacyclic cyanoenones *in vitro* [19, 21, 134], and molecular docking studies using a model for the intervening region of KEAP1 and the C1 of the potential alkylation site of the triterpenoid CDDO-methylamide positioned against C226 of KEAP1 have suggested that C226 may be a target for this triterpenoid [164].

Using ectopically expressed KEAP1 in cultured mammalian cells exposed to a panel of inducers, McMahon and colleagues found that C151 and C288 of KEAP1 each form a distinct sensor with C151 responding to nitric oxide and C288 to alkenals, such as acrolein and 4-hydroxy-2-nonenal [71]. In addition, a third sensor was also identified which is formed by H225, C226, and C613 and was named the Zn^++^ sensor. A molecular model of the BTB domain of KEAP1 was constructed which revealed that C151 is in close spacial proximity with four basic amino acids, that is, K131, R135, and K150, and H154, an environment that could favor the thiolate formation at physiological pH, thereby increasing the cysteine reactivity [71, 81]. In strong agreement, when K131, R135, K150 were substituted with methionine residues, this mutant of KEAP1 had much lower sensitivity to sulforaphane and tBHQ, and was almost completely unresponsive to nitric oxide [71]. In parallel, Fourquet et al. reported that exposure to hydrogen peroxide, to the nitric oxide donor spermine NONOate, to hypochlorous acid, or to *S*-nitrosocysteine causes formation of an intermolecular disulfide bridge linking two KEAP1 monomers via C151 [81]. It has also been suggested that one consequence of the binding of inducers to C151 may be the introduction of a steric clash with amino acid residues in the adjacent *α*-helix which could alter the interaction between KEAP1 and CUL3 [165]. 

**Figure 3:**
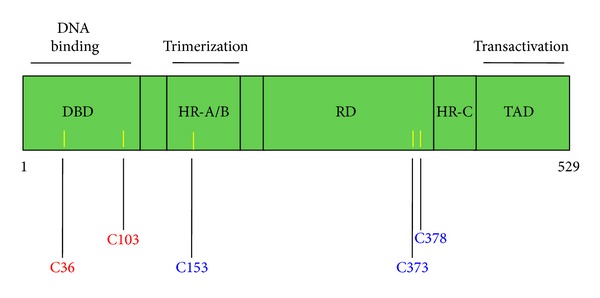
Domain structure of human HSF1. DBD: DNA-binding domain (amino acids 1–110); HR-A/B: heptad repeat regions A and B (amino acids 130–203); RD: regulatory domain (amino acids 221–383). This domain negatively regulates the transactivating activity of HSF1; HR-C: heptad repeat region C (amino acids 384–409); TAD: transactivation domain (amino acids 410–529). HSF1 trimerizes through intermolecular interactions between the HR-A/B regions. Trimerization is negatively regulated by intramolecular interactions between the HR-A/B heptad repeat region and the HR-C domains within the monomer. The positions of the cysteine residues are indicated with yellow bars. The cysteine residues which participate in activating intermolecular disulfide bonds are shown in red, and those which participate in inactivating intramolecular disulfide bonds are shown in blue.

The finding that similar to the KEAP1/NRF2 pathway, sulfhydryl reactivity of small molecule inducers is important for activating the heat shock response is a much more recent discovery, and the identities of the sensor proteins are not firmly established. Depending on the biological system, cysteine-containing HSF1 or its negative regulators, Hsp90 or Hsp70, could potentially play this role. The HSF1 monomer has several distinct domains [61] (Figure 3): (i) DBD: N-terminal DNA-binding domain (amino acids 1–110); (ii) HR-A/B: heptad repeat regions A and B (amino acids 130–203); (iii) RD: centrally located regulatory domain which negatively regulates the transactivating activity of HSF1 (amino acids 221–383); (iv) HR-C: heptad repeat region C (amino acids 384–409); and (v) TAD: C-terminal transactivation domain (amino acids 410–529). HSF1 trimerizes through intermolecular interactions between the HR-A/B regions, whereas trimerization is negatively regulated by intramolecular interactions between the HR-A/B heptad repeat region and the HR-C domains within the monomer. Mammalian HSF1 contains five conserved cysteine residues. It has been shown that activation of murine HSF1 by H_2_O_2_ is dependent on C35 and C105, which are located within the DNA-binding domain of the transcription factor and form a disulfide bridge in response to stress [82]. In the human HSF1, the corresponding pair of cysteines (i.e., C36 and C103) have been shown to form an intermolecular disulfide bond, a process which causes trimerization of the transcription factor and binding to heat shock elements in the promoter regions of heat shock genes [83]. In contrast, the formation of an intramolecular disulfide bond (in which C153, C373, and C378 participate) inhibits trimerization and DNA binding. These results are in agreement with earlier studies which showed that single mutation of C153 or double substitutions of C373 and C378 with serine residues prevented the formation of oxidized HSF1 in response to diamide [114], whereas treatment of HeLa cells with the glutathione-depleting agent L-buthionine sulfoximine promoted the formation of this oxidized species of HSF1 and blocked the heat-induced DNA binding activity of HSF1 [166].

**Figure 4:**
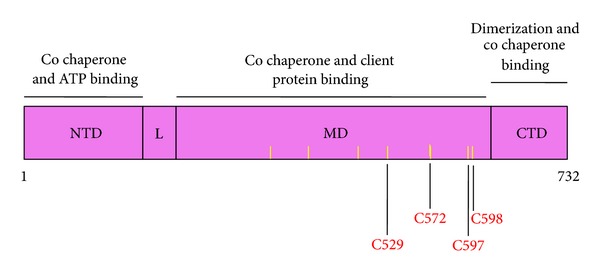
Domain structure of human Hsp90*α*. N-terminal domain (amino acids 1–209). This is the major site of ATP binding; L: flexible linker region (amino acids 210–271); MD: middle domain (amino acids 272–628) through which Hsp90 interacts with many of its client proteins; CTD: C-terminal domain (amino acids 629–732). Hsp90 forms a homodimer through the CTD. The positions of the cysteine residues are indicated with yellow bars. The most commonly modified cysteine residues by sulfhydryl-reactive small molecules are shown in red.

In addition or alternative to directly modifying cysteine residues within HSF1, sulfhydryl-reactive activators may also react with cysteine residues of its negative regulators, Hsp90 and/or Hsp70. Inhibition of the chaperone activities of Hsp90 and/or Hsp70 may lead to the release of HSF1 and activation of the heat shock response. Indeed, induction of the heat shock response occurs commonly when Hsp90 activity is inhibited. Human Hsp90 is a homodimeric multidomain protein which contains four distinct regions: (i) N-terminal domain (amino acids 1–209) where ATP binds; (ii) a charged flexible linker region (amino acids 210–271); (iii) middle domain (amino acids 272–628) where Hsp90 interacts with many of its client proteins; and (iv) C-terminal domain (amino acids 629–732) through which the protein dimerizes (Figure 4) [167, 168]. The C-terminal domain contains a conserved MEEVD amino acid sequence implicated in binding to cochaperones with tetratricopeptide repeat (TRP) domains [169, 170]. Posttranslational modifications of Hsp90 such as acetylation [171], phosphorylation [172, 173], and *S*-nitrosylation [174–177] have all been implicated in the regulation of the activity of the chaperone. Four of the seven cysteine residues of the mammalian Hsp90 are of particular importance for its chaperone function. It has been shown that in human Hsp90*α*, *S*-nitrosation at C597 inhibits the ATPase activity of the chaperone [177]. C521 and C589/590 in the rat Hsp90*β*, corresponding to C529 and C597/C598 in Hsp90*α*, are highly reactive [178]. Substituting C597 in human Hsp90*α* with *S*-nitrosation-mimicking residues, such as asparagine and aspartic acid, shifts the conformational equilibrium of the protein towards the open conformation, thus decreasing its chaperone activity [179]. This conclusion is also supported by molecular dynamics simulation approaches which have indicated that C597 is involved in regulating the conformation in Hsp90 [180]. Whereas C597 is conserved in eukaryotes and some bacteria, in yeast Hsp90, which is devoid of any cysteine residues, the corresponding amino acid is A577, and mutation to asparagine at this site strongly impairs the N-terminal association after ATP binding [179]. 

Recombinant human Hsp90*α* has been shown to be modified by 4-hydroxy-2-nonenal at C572 [181], and C521 in recombinant Hsp90*β* forms a thiocarbamoylation conjugate with 6-methylsulfinylhexyl isothiocyanate [182]. Endogenous Hsp90 is also among the proteins that were found to be modified by 4-hydroxy-2-nonenal [72, 183] and by its azido- and alkynyl-tagged derivatives as identified by the use of click chemistry and ex vivo biotinylation [184]. In addition, modifications in Hsp90 by 4-hydroxy-2-nonenal have been reported to occur in liver in a rat model of alcoholic liver disease [185]. By the use of proteomic and click chemistry approaches, Hsp90 was identified as being modified when HEK293 cells were exposed to the sulfoxythiocarbamate derivative of sulforaphane, STCA [86, 93]. Sulforaphane was also recently shown to modify Hsp90 *in vitro* and to disrupt the interaction between Hsp90 and its co-chaperone Cdc37 and cause degradation of Hsp90 client proteins in pancreatic cancer cells [186]. Notably, in addition to reacting with cysteine, electrophilic compounds such as 4-hydroxy-2-nonenal can also react readily with histidine residues. Thus, the use of noncovalent affinity capture with a biotinyl-geldanamycin probe isolated both isoforms of the Hsp90*α* and Hsp90*β* from human RKO colorectal cancer cells and enabled the detection of histidine adducts that were endogenously formed by the treatment with 4-hydroxy-2-nonenal [187]. Interestingly, no cysteine adducts on either Hsp90*α* or Hsp90*β* were observed in this study.

**Figure 5:**
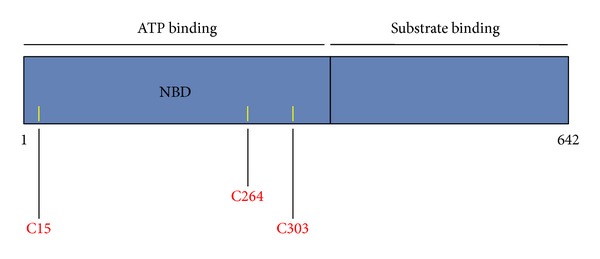
Domain structure of yeast Ssa1 Hsp70. The N-terminal half of the protein formS the nucleotide binding domain, and the C-terminal half is the substrate binding domain. The cysteine residues are indicated with yellow bars and their positions are shown in red.

HSF1 is also negatively regulated by Hsp70 and Hsp40. It is thus possible that structural alterations within these negative regulators may lead to the activation of HSF1 and induction of the heat shock response. Interestingly, modifications of both Hsp90 and Hsp70 are relatively commonly found after exposure to electrophiles. Hsp70 was found to be modified in cells which had been exposed to 4-hydroxy-2-nonenal [72, 183] as well as to its azido- and alkynyl-tagged derivatives [184]. Hsp70 was also identified as one of the proteins that were modified by 4-hydroxy-2-nonenal in rat liver following chronic ethanol treatment [185]. Immunoprecipitation experiments have demonstrated that 4-hydroxy-2-nonenal disrupts the interaction of ectopically expressed c-Myc-tagged Hsp70 with HSF1 and induces the heat shock response [72]. Most recently, the Hsp70 chaperone Ssa1 was identified as the sensor for a range of sulfhydryl-reactive electrophiles which trigger the HSF1-mediated heat shock response in yeast (*Saccharomyces cerevisiae*) [73]. Notably, in yeast only Hsp70, but not Hsp90 or HSF1, contains cysteine residues: C15, C264, and C303, all of which are located within the nucleotide binding domain of Hsp70 Ssa1 (Figure 5). All three cysteines in Ssa1 can be modified by the alkylating agent *N*-ethylmaleimide, causing conformational changes and disruptions in interdomain communication, and ultimately resulting in inhibition of the ATP binding ability and protein folding activity of the chaperone [188–190]. Serine substitutions of C264 and C303, either individually or in combination, rendered this Ssa1 mutant unresponsive to celastrol, cadmium, and 4-hydroxy-2-nonenal, but interestingly, did not affect cellular responsiveness to thermal stress [73], indicating that the mechanisms for activation of HSF1 by sulfhydryl reactive agents and by thermal stress may not be the same. The same study showed that treatment of FLAG-tagged Ssa1-expressing cells with an alkyne derivative of 4-hydroxy-2-nonenal resulted in labelling of the wildtype Ssa1 by the electrophilic alkyne. In contrast, labelling was almost completely abolished in cells expressing the double C264S/C303S mutant and was decreased very substantially for the C303S mutant, suggesting that C303 is highly reactive and might be the prime site of electrophilic modification. Unlike the wildtype protein, a mutant in which C264 and C303 were substituted with aspartic acid, which mimics the sulfinic acid oxidation form of cysteine, failed to complement the HSF1 derepression phenotype of cells deficient for Ssa1. Taken together, these findings indicate that modification of C264 and C303 may result in structural alterations of Ssa1 ultimately leading to loss of its HSF1 repressor function. Mammalian Hsp70 was recently shown to be oxidized and inactivated by the redox-active compound, methylene blue at C306, and molecular modeling has suggested that oxidation of C306 also exposes C267 to modification, and it was proposed that both events contribute to loss of ATP binding and chaperone activity [191].

In addition to directly affecting the immediate repressing partners of HSF1, electrophilic HSF1 activators may also operate indirectly, by chemically modifying and thus altering the function of co-chaperones that form the Hsp90 complex machinery. Experimental evidence suggests that this could be the case for the co-chaperone Cdc37, which is involved in delivering client proteins to the Hsp90 complex. Cdc37 binds as a dimer to the N-terminal domain of Hsp90 and arrests the ATPase cycle of the chaperone during client-protein loading [192, 193]. The interaction between the ^1^H,^15^N-labeled N-terminal domain of Hsp90 with unlabeled full-length Cdc37 has been examined in the absence of presence of celastrol using Heteronuclear Single Quantum Coherence (HSQC) NMR spectroscopy [194]. In combination with mutagenesis analysis and chemical modification (with *N*-ethylmaleimide) of the 9 cysteine residues of Cdc37, it was found that celastrol reacts with the 3 cysteine residues within the N-terminal domain of Cdc37. This causes conformational changes in both the N-terminal and in the middle Hsp90-binding domains of Cdc37, and ultimately disrupts the Cdc37-Hsp90 complex.

## 6. Cross-Talk between the KEAP1/NRF2 Pathway and the Heat Shock Response

The KEAP1/NRF2 pathway and the heat shock response are two separate cytoprotective pathways. Induction of the NRF2-dependnet target enzyme NQO1 by sulfhydryl-reactive agents of different structural types requires NRF2, but not HSF1, whereas induction of the HSF1-dependent protein Hsp70 by the same compounds depends on the presence of HSF1, but is independent of NRF2 [86]. Nonetheless, there is cross-talk between the two pathways. One example is rodent heme oxygenase 1, also known as Hsp32, which is induced both through activation of the KEAP1/NRF2 pathway and by heat shock [38, 195–199]. Interestingly, the presence of ARE sequences has been detected in the promoter regulatory regions of both murine and zebrafish *hsp70 *[129]. Another recently reported intriguing link between the two pathways is the observation that Hsp90 and KEAP1 interact upon heat shock, leading to activation of NRF2 [200]. It is possible that by binding to KEAP1, Hsp90 protects the electrophilic stress sensor from potential damage during heat shock. It will be interesting to determine whether activation of NRF2, as a consequence of the interaction between KEAP1 and Hsp90 during heat shock, might be a contributing factor to the protective effect of heat shock against subsequent exposures to other forms of lethal cell stress.

## 7. Concluding Remarks

It should be noted that *in vitro* cysteine modifications of recombinant versions of the proteins that are targeted by sulfhydryl-reactive agents do not always correlate with the observed modifications in cellular protein targets isolated by the use of affinity probes. In addition, many compounds that react with sulfhydryl groups cause transient depletion of the intracellular glutathione and thioredoxin pools. It is therefore possible that it is the failure to maintain the physiological redox state of critical regulators, such as KEAP1, HSF1, Hap90, and/or Hsp70, rather than, or in addition to, their direct modifications by electrophilic small molecules, that activates the KEAP1/NRF2 pathway and the heat shock response. In the case of KEAP1, this possibility is highly unlikely as some inducers are active at concentrations in the low- and even subnanomolar range [19, 134], whereas glutathione is present in the cell at millimolar concentrations. Interestingly, the concentrations of sulfhydryl-reactive agents which induce the heat shock response are much higher than those that induce the KEAP1/NRF2 pathway. Thus, the possibility for such an indirect effect leading to the activation of HSF1 is more likely. However, it is also possible that the underlying reason for the different concentration requirements for the activation of the two pathways is the difference in the abundance of the protein targets. KEAP1 is a protein of an extremely low abundance. In sharp contrast, Hsp90 is highly abundant and constitutes 1-2% of the total cellular protein in a homeostatic unstressed cell. Another reason for the difference in the inducer concentration requirements could be a difference in the relative nucleophilicity of the cysteine residues of the target proteins and their accessibility for reaction with the electrophilic inducers. It could be proposed (Figure 1) that, at low concentrations, inducers are sensed first by the highly reactive cysteine residues of KEAP1, activating the KEAP1/NRF2 pathway. At higher concentrations, inducers affect the function of other targets, such as HSF1, Hsp90, Hsp70, or a co-chaperone within the Hsp90 complex machinery, leading to the activation of HSF1 and induction of the heat shock response. Future experiments are needed to test this proposal. However, it is clear at present that reactivity with sulfhydryl groups is a major determinant of the mechanism of action of small molecules dual activators of the KEAP1/NRF2 pathway and the heat shock response.

## Figures and Tables

**Table 1:** Examples of small molecules—dual activators of transcription factors NRF2 and HSF1.